# Nurses in Combination

**Published:** 1919-03-15

**Authors:** 


### THE MATRONS' AND SISTERS' DEPARTMENT.

### NURSES IN COMBINATION.

There is an incurable tendency towards impatience in the human mind which often produces disaster at the moment when victory is at hand. It seems men can be patient under adversity, but when the clouds begin to break they can contain themselves no longer. They must have instant realisation of all for which they have been striving, and those who have borne the brunt of the battle must bear also the unjust reproaches of the impatient.

Something like this is happening in the nursing world. A few impatient nurses—they are always the noisy ones—are clamouring for immediate fruits of victory. Regardless of the rising tide, they point to the waste of still uncovered shore, and chafe at the long delay in bringing reforms into every corner of the nursing world. They point to achievements brought about by trades-union action in mechanical employments, and clamour for a trades union of nurses. They fail to comprehend the essential difference between a nurses' trades union and a college of nursing, and stumble because they take short views and attempt short cuts.

Wherever a trades union of nurses has been attempted it has always shown certain marked characteristics. It has been formed of a group of nurses in some particular locality possessed by a sense of obvious and galling grievances. Most of these are probably not nurses in any real sense, but young women in the pupil stage, without power to discriminate between the hardships incidental to their calling and those which are imposed by bad management. They press for definite alleviations without attempting to gauge difficulties. Such a group united under a plausible leader might contend for a shortening of the period of training, as has been done in Melbourne, might desire easier examinations, less theoretical study, less thorough grounding in domestic matters. So long as they got a higher wage and a reduction of hours they would be reckless about the effect of the changes they advocated on their future. They might do much harm and injure their own prospects, but they seldom succeed in making much impression because the conditions of nursing are too complicated for grievances to be dealt with in this blunderbuss style. In one or two notorious instances probationer nurses may have secured by threats boons which would have been freely granted them had they used a more decent method to urge their claims. Are not such boons bought too high? The price is the risk of setting a black mark against their names to the end of their career—a mark such as may not be visible to employers, but is the cause of secret remorse as the enormity of the transgression becomes apparent in later years to the nurse herself.

The only possible way in which to bring about a reform of the conditions under which nurses are trained is to set the matrons and sisters engaged in training nurses to find the remedy. There is no short cut to the redress of abuses which have been handed down from the past, the offspring of obsolete social conditions, and are kept alive in a thousand independently-governed institutions mainly because no one has had the wit to devise means for their extinction. It has taken a long time to shed light into this dark labyrinth. But each matron, each sister who catches the spirit of reform becomes a lantern-bearer, and the darkness is fleeing fast. Week by week we have the pleasure of spreading news of reforms. In London and in the provinces, in old historic establishments and in newly-built little ones, the light is spreading. And one immediate cause of this new spirit is, doubt it not, the closer co-operation between matrons, sisters, and nurses brought about through the College and its centres. When we look back and count the achievements of the last two years we are amazed. It was but at the last annual meeting of the College that Miss Baillie announced the inauguration of a one day's rest in seven for her nurses at Bristol. And already the one day in seven is become the watchword of progress, and is an accomplished fact in many directions. Hardly a hospital in the kingdom but has made substantial additions to the nurses' pay. Legislation for nurses is at last on the eve of accomplishment. The College has won its endowment. Its centres are soundly based north, south, east, and west throughout Great Britain. Its educational programme nears completion. Its means of succouring the sick and friendless nurse are secured. Why then this idle talk of Trades Unionism? What the College of Nursing has already achieved is what no trades union has ever been able to effect for its members. Strengthen it further by the adhesion of a few thousand more members, and the nursing profession will have an engine powerful enough to sweep all obstructions from the path.

